# Reduction of Early‐Onset Calcium Hydroxylapatite‐Carboxymethylcellulose Accumulations Using Focused Mechanical Vibration: An Experimental Model and Case Report

**DOI:** 10.1111/jocd.70272

**Published:** 2025-07-17

**Authors:** Jaya Krishna Rose Batra, Brandi Gregge, Alec D. McCarthy, W. Gregory Chernoff, Shino Bay Aguilera, Sanjay Batra

**Affiliations:** ^1^ VIAS Partners Doylestown Pennsylvania USA; ^2^ Mint & Needle Middletown Delaware USA; ^3^ Merz Aesthetics Global Medical Affairs Raleigh North Carolina USA; ^4^ Chernoff Cosmetic Surgery Indianapolis Indiana USA; ^5^ Shino Bay Cosmetic Dermatology & Laser Institute Fort Lauderdale Florida USA

**Keywords:** clinical reseach, complications, granuloma, hand rejuvenation, microdermabrasion, research

## 1 Introduction

Particle‐containing biostimulatory treatments, such as calcium hydroxylapatite‐carboxymethylcellulose (CaHA‐CMC; Radiesse, Merz North America Inc., Franksville, WI, USA), have become increasingly popular for their ability to restore soft tissue volume and promote extracellular matrix regeneration [1, 2]. Despite their clinical effectiveness, complications like noninflammatory nodules can occur, primarily attributed to localized focal accumulations (FAs) of particles rather than immune‐mediated inflammatory responses [3]. These nodules typically result from superficial injections, accidental boluses, injection of large volumes of highly concentrated products, or poor dispersion of the material during injection [4]. FAs can arise minutes to days postinjection [3]. In contrast, delayed‐onset nodules, which are generally driven by immune responses, often appear months after injection [5].

Previous strategies to manage these nodules have ranged from conservative measures, such as manual massage and warm compresses to more aggressive pharmacologic interventions, ablative technologies, and even negative pressure aspiration, with varying degrees of efficacy [6, 7, 8, 9]. Recently, structured algorithms categorizing these interventions based on their invasiveness, effectiveness, and patient burden have been proposed [3]. Specifically, Level 1 interventions, which emphasize minimally invasive dispersion techniques, have shown promise in effectively resolving these accumulations by redistributing the filler particles over a broader tissue area.

**FIGURE 1:**
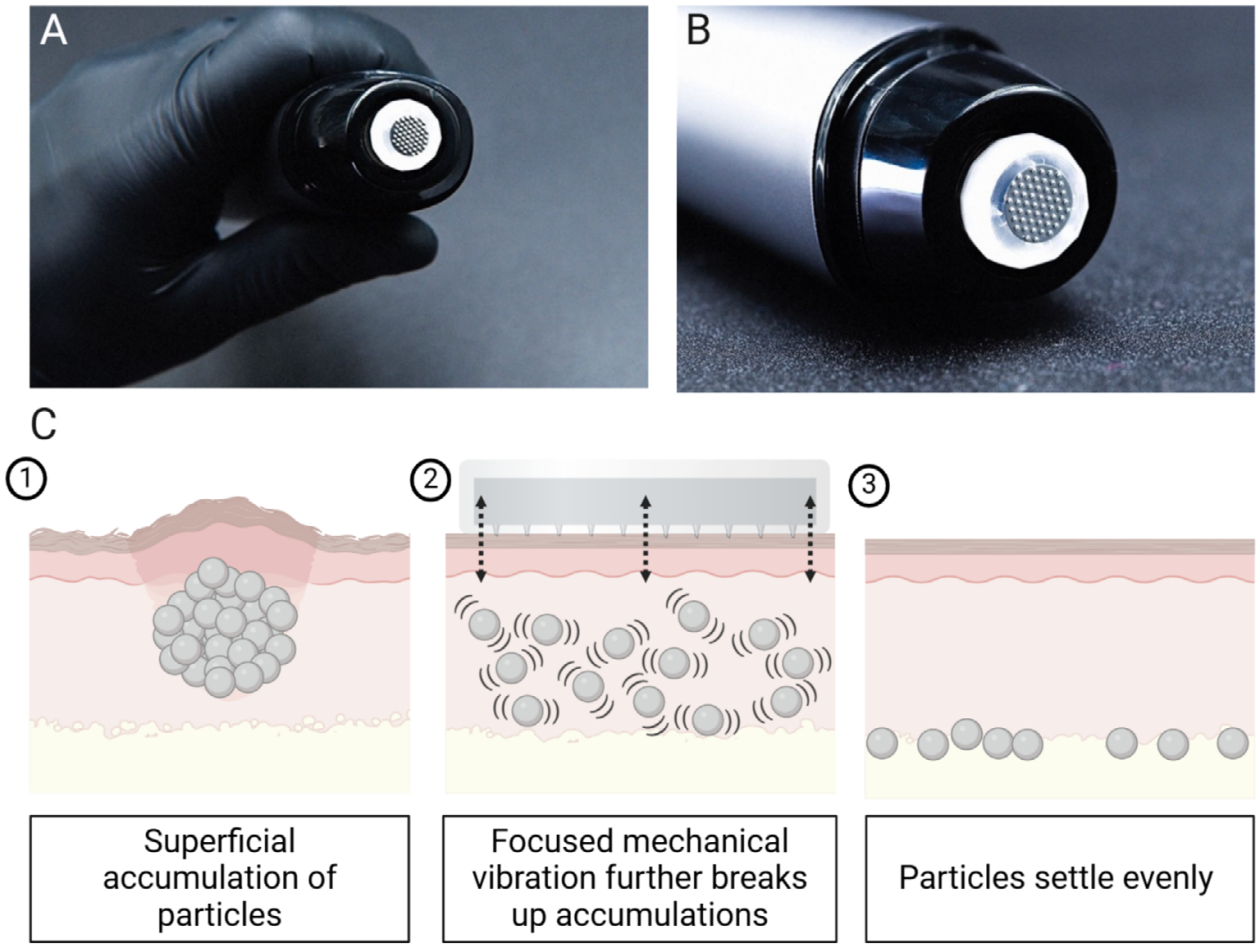
(A) Images of the nanochip vibrational device (B) and the 30‐array tip in high magnification. (C) The proposed mechanism of focused mechanical vibration consists of using focused mechanical energy to disperse particles into surrounding soft tissue.

Building upon these principles, focused mechanical vibration (FMV) has emerged as a novel, minimally invasive intervention [10]. Similar in concept to lithotripsy in urology, FMV utilizes targeted vibrational energy to disrupt and disperse filler accumulations. Our prior work demonstrated that FMV combined with aqueous dispersion successfully resolved superficial CaHA nodules rapidly and effectively. This current preclinical evaluation and case report introduces the use of a vibrational nanochip device (Juvasonic, VIAS Partners, New Hope, PA, USA), which applies percussive mechanical vibration directly to the nodules without penetrating the skin with microneedles (Figure 1A,B). The energy exerted by the device is proposed to directly disperse accumulated particles into surrounding soft tissue (Figure 1C).

The objective of this study is to provide a preliminary in vitro assessment and clinical case report of FMV to smooth early‐onset CaHA accumulation using a percussive nanochip device. This report aims to further validate FMV as a reliable first‐line treatment modality for noninflammatory nodules, FAs, and smoothing of superficial lumps.

## 2 Methods

### 2.1 Preclinical Assessment

A skin‐mimicking hydrogel (Skintegrity, Medline Industries Inc., Northfield, IL, USA) was used to simulate the mechanical response of skin to vibrational energy. The hydrogel was injected with 0.2 mL of undiluted CaHA‐CMC to represent a nodule. A percussive microneedling device (Juvasonic, VIAS Partners, Doylestown, PA, USA) was operated at 8800 RPM (the fastest setting) with a tip attachment consisting of 30 protrusions that have a depth of 0.25 mm. The hydrogel with embedded CaHA‐CMC was photographed on a black background prior to treatment and 15 and 30 s after vibration was applied. The images underwent color deconvolution to isolate the CaHA‐CMC, and the magic wand tool was used to highlight the entire nodule for morphological characterization. Vibrational energy was applied for 30 s. Morphological changes were quantified by tracking nodule area, aspect ratio (major axis/minor axis), and major axis length over time using macroscopic imaging assessed with ImageJ based on a similar previous protocol [11].

## 3 Results

### 3.1 Hydrogel Study

**FIGURE 2:**
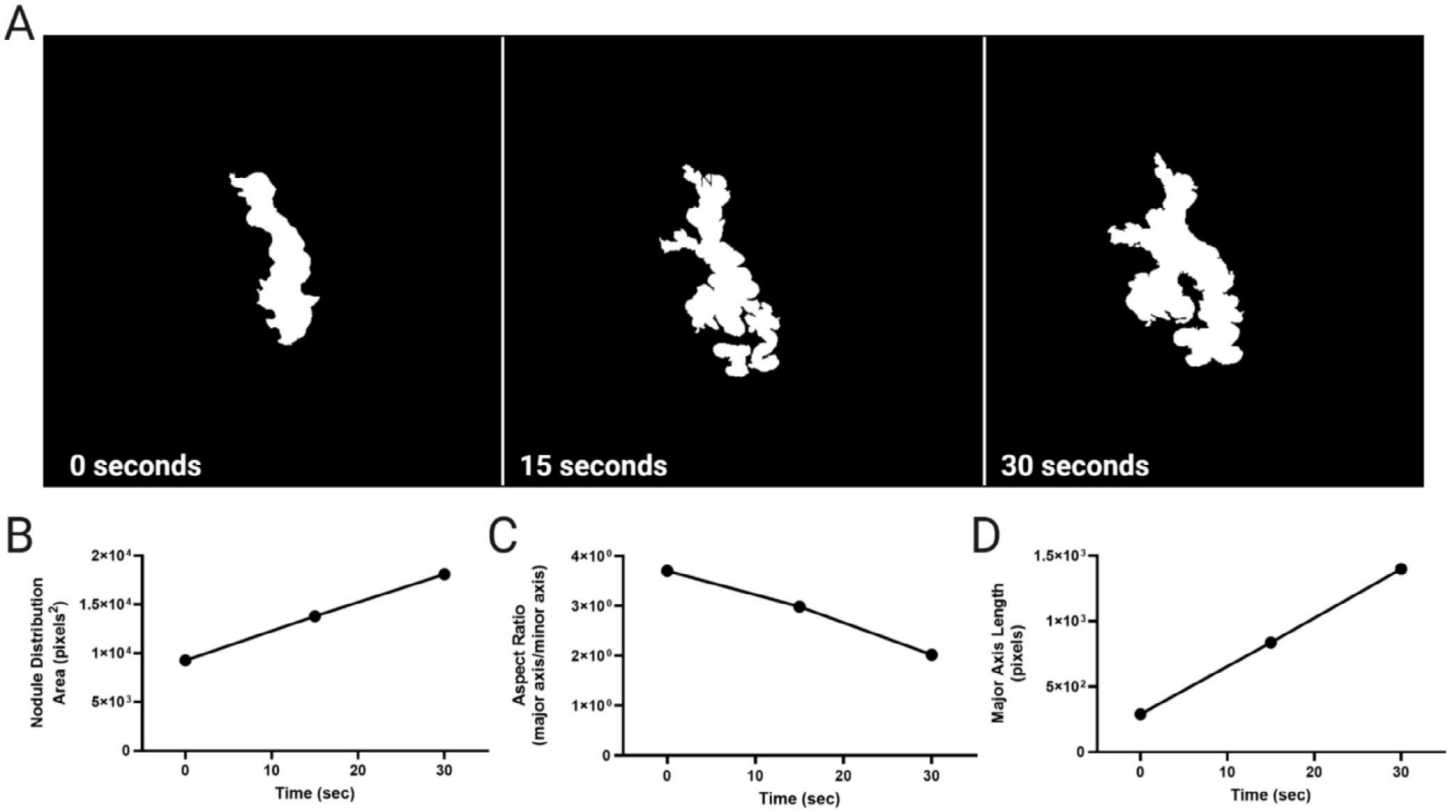
Images of the hydrogel in vitro model showing (A) gradual distribution of the CaHA microspheres, (B) increase in the lateral distribution area, (C) decrease in the aspect ratio, and (D) increase in the major axis length following 15 and 30 s of focused mechanical vibration.

The preclinical hydrogel model demonstrated significant particle dispersion under vibrational treatment (Figure 2A). The nodule area increased linearly from approximately 9266 pixels^2^ at baseline to 18 091 pixels^2^ after 30 s (Figure 2B). Due to the nearly incompressible nature of the hydrogel (as dictated by Poisson's ratio), an observed increase in nodule inherently corresponds to a reduction in nodule height. Supporting this concept, the aspect ratio decreased from 3.7 to 2.0, indicating a shift from an elongated form to a more isotropic shape, consistent with lateral spreading (Figure 2C). Furthermore, the major axis length increased linearly from approximately 290.1 to 1398.8 pixels, suggesting a substantial and time‐dependent horizontal elongation and redistribution of particles that further confirm a concurrent vertical flattening of the nodule (Figure 2D). These parameters exhibited clear dependence on vibration exposure duration. Collectively, these observations highlight that vibrational energy facilitates lateral strain, reduces axial strain, and ultimately smooths the nodule's surface.

### 3.2 Clinical Case

**FIGURE 3:**
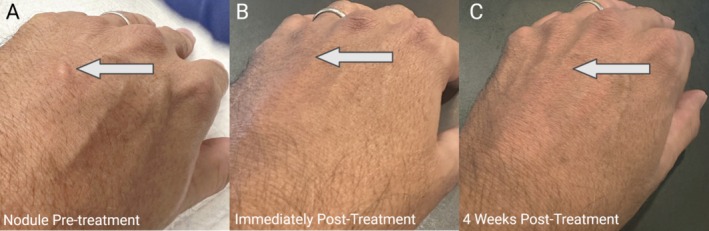
Clinical photos of the nodule on the hand of a 61‐year‐old male patient (A) before intervention, (B) immediately after focused mechanical vibration was applied, and (C) 4 weeks after treatment, marking total resolution.

A 61‐year‐old male patient presented with a single, nontender, palpable, and visibly detectable nodule approximately 3 h after receiving an injection of undiluted CaHA‐CMC (+) to the dorsum of the hand (Figure 3A). Although not painful, the nodule, measuring approximately 0.2 mL, was aesthetically displeasing to the patient and a Level 1 intervention, FMV, was pursued according to a previously reported algorithm [3]. A percussive, nanochip device (Juvasonic) was used to apply FMV. The device was positioned perpendicular to the skin and passed over the nodule approximately 20 times and for approximately 2 min using an uncrosslinked hyaluronic acid topical serum (Maskād Peptide Serum, WeThrivv, Doylestown, PA) as a gliding agent. Unlike previous reports using FMV, no saline was injected into the nodule prior to the application of FMV. Mild erythema was observed immediately after the application of FMV. Immediately after FMV, the nodule size decreased by approximately 95% (Figure 3A). Within 24 h, the nodule was completely resolved. Four weeks after nodule treatment, the patient did not observe any return of nodularity (Figure 3C). The patient reported improvements in skin quality within the treatment area and could still manually detect the presence of the CaHA‐CMC but noted that the nodule did not return and that the injected CaHA appeared to reside deeper in the dermis. Written and informed consent was obtained for the patient whose case was reported in this commentary.

## 4 Discussion

The results highlight the efficacy of a vibrational nanochip device in dispersing accumulated CaHA‐CMC microspheres and smoothing irregularities without the invasiveness of mechanical microneedles. The principles of vibration‐induced dispersion are analogous to lithotripsy, where energy disrupts particle aggregates without damaging surrounding tissues [12]. In the preclinical hydrogel model, increased lateral spread and reduced height illustrate the redistribution of particles facilitated by vibrational energy and corroborate previous studies demonstrating similar dispersing phenomena [10].

The clinical application further validated these findings. Immediate reductions in nodule size and effective smoothing through a low‐cost, low‐risk intervention demonstrate the practical benefits of this approach. Further, the noninvasive nature of this protocol offers significant advantages over invasive strategies, such as ablative or surgical techniques. Unlike pharmacologic agents, vibrational energy addresses particle aggregation directly and mechanically, without the risk of systemic drug‐related reactions.

Despite these promising results, several limitations warrant discussion. This study focuses exclusively on very early onset accumulations (within minutes to hours postinjection), so the applicability of FMV to later‐stage nodules developing days or weeks after injection remains unknown. The preclinical model, whereas effective for imaging, does not fully replicate the complexities of human tissue over time. Additionally, the long‐term stability of the dispersed particles and the durability of cosmetic outcomes require further study. Future research should focus on optimizing device parameters and evaluating outcomes in larger patient cohorts and in nodules with longer residence times.

## 5 Conclusion

The investigated vibrational nanochip device, band name JUVASONIC offers a noninvasive option for managing immediate onset noninflammatory CaHA nodules. By leveraging vibrational energy, this method effectively redistributed particles, reduced nodule height, and smoothed tissue contours. The use of vibrational dispersion provides a promising approach to nodule resolution.

## Author Contributions

**Jaya Krishna Rose Batra:** conceptualization, data curation, investigation, methodology, writing – review and editing. **Brandi Gregge:** data curation, investigation, resources, methodology, visualization, writing – review and editing. **Alec D. McCarthy:** conceptualization, formal analysis, writing – original draft, writing – review and editing, visualization, methodology. **W. Gregory Chernoff:** conceptualization, supervision, writing – review and editing. **Shino Bay Aguilera:** conceptualization, validation, methodology, writing – review and editing. **Sanjay Batra:** conceptualization, methodology, software, validation, formal analysis, investigation, resources, data curation, writing – review and editing, supervision, project administration. All authors have read and approved the final manuscript.

## Conflicts of Interest

The authors declare no conflicts of interest.

## Data Availability

The data that support the findings of this study are available from the corresponding author upon reasonable request.
